# Change in Atrial Fibrillation Burden over Time in Patients with Nonpermanent Atrial Fibrillation

**DOI:** 10.1155/2020/9583409

**Published:** 2020-04-17

**Authors:** Philipp Krisai, Stefanie Aeschbacher, Matthias Bossard, Elena Herber, Steffen Blum, Pascal Meyre, Thilo Burkard, Michael Kühne, Stefan Osswald, Beat A. Kaufmann, David Conen

**Affiliations:** ^1^Department of Cardiology, University Hospital Basel, Basel, Switzerland; ^2^Cardiovascular Research Institute Basel, University Hospital Basel, Basel, Switzerland; ^3^Cardiology Division, Heart Center, Luzerner Kantonsspital, Luzern, Switzerland; ^4^Medical Outpatient and Hypertension Clinic, ESH Hypertension Centre of Excellence, University Hospital Basel, Basel, Switzerland; ^5^Population Health Research Institute, McMaster University, Hamilto, Canada

## Abstract

**Introduction:**

The natural course of atrial fibrillation (AF) is not well defined. We aimed to investigate the change in AF burden over time and its associated risk factors among AF patients.

**Methods:**

Fifty-four participants with recently documented paroxysmal or persistent AF were enrolled. Main exclusion criteria were permanent AF or previous catheter ablation for AF. AF burden was calculated as time in AF divided by total recording time using yearly continuous 7-day Holter-ECG recordings. A relative change ≥10% or an absolute change >0.5% in AF burden between two yearly Holter-ECG recordings was considered significant.

**Results:**

Mean age was 67 years, 72% were men. The proportion of patients with no recorded AF increased from 53.7% at baseline to 78.6% (*p*=0.1) after 4 years of follow-up. In 7-day Holter-ECG recordings performed after baseline, 23.7% of participants had a decrease and 23.7% an increase in AF burden. In separate mixed effect models, AF burden over time was associated with prior stroke (*β* 42.59, 95% CI (23.40; 61.77); *p* < 0.0001), BNP (*β* 0.05, CI (0.02; 0.09); *p*=0.005) end-diastolic (*β* 0.49, CI (0.23; 0.74); *p*=0.0003) as well as end-systolic (*β* 0.25, CI (0.05; 0.46); *p*=0.02) left atrial volume, left atrial ejection fraction (*β* −0.43, CI (−0.76;−0.10); *p*=0.01), *E*-wave (*β* 36.67, CI (12.96; 60.38); *p*=0.003), and deceleration time (*β* −0.1, CI (−0.16; −0.05); *p*=0.002). In a multivariable model, a history of prior stroke (*β* 29.87, CI (2.61; 57.13); *p*=0.03) and BNP levels (*β* 0.05, CI (0.01; 0.08); *p*=0.007) remained significantly associated with AF burden.

**Conclusions:**

Few patients with paroxysmal or persistent AF have AF episodes on yearly 7-day Holter-ECG recordings, and AF progression is rare. AF burden was independently associated with a history of prior stroke and BNP levels.

## 1. Introduction

Atrial fibrillation (AF) is the most common cardiac arrhythmia, and its incidence is increasing [1, 2]. Patients with AF are at increased risk for death, stroke, and congestive heart failure in comparison to the general population [3–5].

Previous cohort studies have shown progression rates from paroxysmal to persistent AF after first diagnosis in up to 15% after 1 year and in up to 24% after 5 years [6–8]. Possible mechanisms discussed for this progression included left atrial enlargement with fibrosis and impaired calcium handling, though these mechanisms and their influence on progression are still incompletely understood [9–12]. The course of AF is further influenced by comorbidities such as arterial hypertension or thyroid dysfunction, genetics, and psychological factors [13–17]. Nevertheless, clinical classification of AF might not correlate with the actual burden of AF, and a subset of AF patients might even develop a decrease in AF burden [18–20]. Recent studies using cardiac pacemakers or implantable loop recorders showed both an increase and a decrease in AF burden over time [19, 21]. However, patients requiring cardiac pacemakers represent a highly selected cohort and may not reflect the overall population with AF.

Therefore, the aim of our study was to investigate the natural course of AF burden over several years using the 7-day Holter-ECG monitoring and a comprehensive phenotypic assessment, including echocardiography, ambulatory blood pressure, and biomarkers.

## 2. Methods

### 2.1. Study Participants

All study participants included in the current analysis were participants of a single center prospective cohort study (“Basel Atrial Fibrillation Progression Cohort Study”). We enrolled 54 individuals with known paroxysmal or persistent AF who had at least one documented AF episode within 12 months before inclusion. Paroxysmal AF was defined as AF episodes lasting no longer than 7 days with spontaneous conversion, persistent AF as AF with at least one episode >7 days, or the need for cardioversion according to guidelines at the time of study inception [22]. Participants with persistent AF had to be in sinus rhythm at study entry. Main exclusion criteria were permanent AF, scheduled or previous catheter ablation for AF, acute illness at time of enrolment, short, transient forms of AF, or any other severe, life-limiting illness. The study protocol was approved by the local ethics committee and informed written consent was obtained from each participant.

### 2.2. Assessments

Standardized questionnaires were used to assess personal, medical, lifestyle, and nutritional factors. Weight and height were directly measured in a standardized manner, and body mass index (BMI) calculated as body weight in kilogram divided by height in meters squared. The 7-day Holter-ECG recordings (Lifecard CF, Spacelabs) were obtained to calculate the individual AF burden, defined as time in AF divided by total recording time. At least two trained assessors adjudicated the episodes of RR interval instability over at least 30 seconds as AF using a centralized, standardized process. Participants were considered to have AF progression or regression if the AF burden showed an increase or decrease between the first and last 7-day Holter-ECGs, respectively. As to our knowledge, there are no previous data to provide guidance on the definition of a significant change in AF burden, so we prespecified as significant a relative change ≥10% or an absolute change > 0.5%, corresponding to approximately one hour between two yearly Holter-ECG recordings.

Study echocardiograms were obtained by dedicated echocardiographers as previously described [23, 24]. In brief, two-dimensional (2D) and real-time three-dimensional (3D) images were obtained by standardized transthoracic echocardiograms using a Philips iE 33 ultrasound system (Philips Medical Systems, Andover, MA) equipped with an X5-1 transducer. At least two sets of 3D image data per participant were acquired using full volume loops during breath-hold with gated acquisition. All echo data were analysed offline using a dedicated workstation (LV and 4-D LA-analysis, TomTec-Imaging Systems, Unterschleissheim Munich, Germany) by a blinded cardiologist in a standardized manner. Left atrial 3D echo data including volume and ejection fraction of participants in AF were only included if image quality was evaluated as sufficient (84.3%). If a patient was in AF at the time of the echo, parameters of left atrial contraction, such as A-wave or left atrial stroke volume, were not included, but if image quality was sufficient, volume data were used.

Ambulatory blood pressure (BP) measurements were obtained, using a validated automatic device (Mobil-O-Graph PWA, IEM), [25] every 15 minutes from 08 : 00 to 22 : 00 and every 30 minutes in the remaining time period. Individual diaries were used to define awake and asleep BP values. Venous blood samples were drawn from each participant and immediately analysed.

All assessments were repeated during yearly follow-up visits.

### 2.3. Statistical Analysis

Baseline characteristics were stratified by the presence or absence of AF during the baseline 7-day Holter-ECG recording. The distribution of continuous variables was assessed using skewness, kurtosis, and visual inspection of the histogram. Continuous data were presented as means (+/− standard deviations) or medians (interquartile ranges (IQR)), as appropriate, and compared using *t* tests or Wilcoxon rank-sum tests. Categorical variables were presented as counts (percentages) and compared using Fisher exact tests.

Separate multivariable, mixed effects linear regression models were built to investigate the relationship between AF burden as a continuous outcome variable and clinical, laboratory, and echocardiographic parameters as predictor variables. Participant identity numbers were entered in the models as random effects and the duration of follow-up was used as a covariate. Crude models were further adjusted for sex and age. Significantly, associated parameters were then entered in a combined multivariable regression model. Collinearity was assessed for all variables entered in the final model using the variance of inflation factor and inspection of the correlation matrix. If collinearity was detected, the variable with the highest R-square in the univariate model was used and the other excluded.

All statistical analyses were performed using SAS version 9.4 (SAS Institute Inc, Cary, NC) after data collection has been completed. A *p* value of <0.05 was prespecified to indicate statistical significance.

## 3. Results

Baseline characteristics stratified by the presence or absence of AF in the baseline 7-day Holter-ECGs are shown in Table 1. Mean age was 67 years and 72.2% were males. At baseline, no AF burden was recorded in 53.7% of the study population. Compared to participants with no detected AF, there was a higher rate of nonparoxysmal AF (*p*=0.04) and prior strokes (*p*=0.003) as well as a lower intake of beta-blockers (*p*=0.045) in participants with AF burden in the baseline Holter-ECG. There were no significant differences in BP, laboratory measurements, or echocardiographic parameters. The median (IQR) Holter-ECG recording time was 166 (157; 167) hours, and the mean time in AF was 2.3 hours per day among all participants.

**Figure 1:**
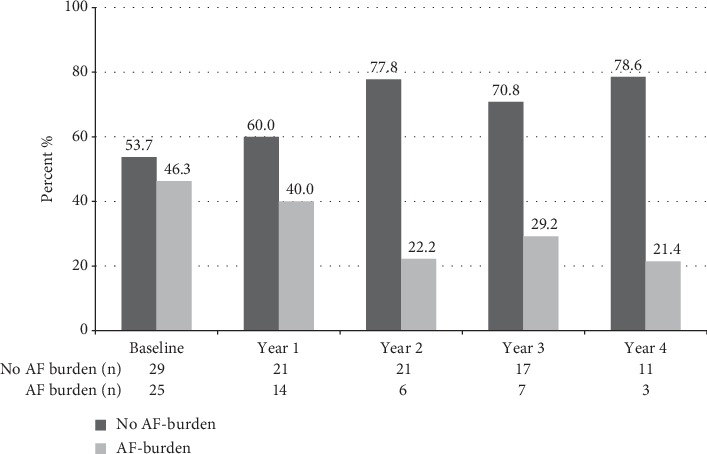
Participants stratified by the presence or absence of atrial fibrillation (AF) over all study visits.

**Figure 2:**
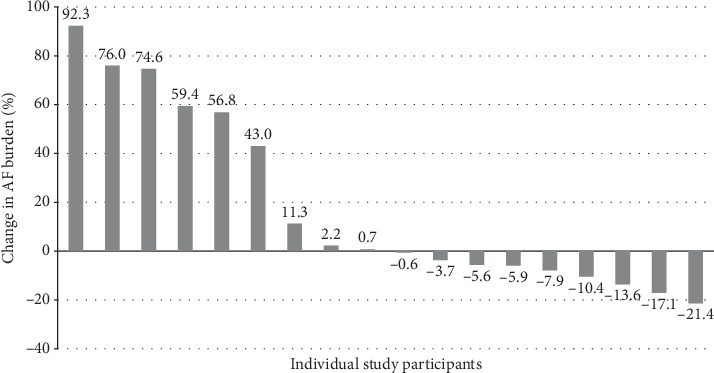
Individual change in atrial fibrillation burden between the first and last Holter-ECG recording. Each bar represents one participant. Only participants with a significant increase or decrease in atrial fibrillation burden are shown.

At least one follow-up was available in 38 (70.4%) patients. The proportion of patients without AF episodes during follow-up Holter-ECG recordings increased to 78.6% (*p* = 0.1) after 4 years of follow-up (Figure 1). 17 (44.7%) patients had no AF burden recorded by their baseline and last Holter-ECG. Nine participants (23.7%) had an increase in AF burden, and three of them (33.3%) developed permanent AF. There was a significant decrease in AF burden over time in nine study subjects (23.7%). Three participants (7.9%) remained stable. The individual changes in AF burden of the 18 patients with significant increases and decreases in AF burden between the first and last Holter-ECG are shown in Figure 2. Of the 14 patients with completed 4-year follow-up, AF was present in 9 (64.3%) and absent in 5 (35.7%) at baseline. Of the 11 patients with no AF at the 4-year follow-up, 6 (54.5%) had AF in the baseline Holter-ECG monitoring. At baseline, participants with a subsequent increase of AF burden had a median AF burden of 5.6% (0; 10.0) compared to 10.4% (5.9; 16.6) in participants with a subsequent decrease in AF burden (*p*=0.2). In the group of patients with an increase in AF burden, one patient experienced a myocardial infarction, and one patient was diagnosed with skin cancer during follow-up. No clinical events were recorded in patients with a decrease in AF burden. In patients without AF burden, one patient was diagnosed with prostate cancer and one patient with deep vein thrombosis. No stroke, new heart failure, systemic embolism, or bypass operation was recorded during follow-up.

In separate random effects models, AF burden was significantly related to a history of stroke (*β* 43.09, CI (23.22; 62.95); *p* < 0.0001), brain natriuretic peptide (BNP) levels (*β* 0.05, CI (0.02; 0.09); *p*=0.005), end-diastolic (*β* 0.49, CI (0.23; 0.74); *p*=0.0003) as well as end-systolic (*β* 0.25, CI (0.05; 0.46); *p*=0.02) left atrial volume, left atrial ejection fraction (*β* −0.42, CI (−0.76; −0.09); *p*=0.01), *E*-wave (*β* 37.93, CI (13.98; 61.89); *p*=0.002), and deceleration time (*β* −0.1, CI (−0.16; −0.05); *p*=0.0002) (Table 2). In a combined multivariable linear regression model, a history of a prior stroke (*β* 29.87, CI (2.61; 57.13); *p*=0.03) and BNP levels (*β* 0.05, CI (0.01; 0.08); *p*=0.0007) remained significantly associated with AF burden (Table 3). Due to collinearity between left atrial ejection fraction and end-diastolic and end-systolic volume, the latter two were not considered for the combined model.

## 4. Discussion

To the best of our knowledge, this is one of the first prospective studies to assess the development of AF burden over several years using repeated 7-day Holter-ECG monitoring. The present analysis indicates that in more than half of all participants with recently documented paroxysmal or persistent AF, no AF episodes could be recorded using the 7-day Holter-ECG monitoring. This proportion increased over time. A similar proportion of patients had either an increase or decrease in AF burden over time.

Based on prior studies using medical records for AF type assessment, the natural course of AF was believed to be primary progressive with no or little disease regression [6–8]. More recent data in patients with implanted cardiac devices showed a high variability in AF burden over time. Both an increase and a decrease in AF burden was observed [19]. Our study confirms these results and extends these findings to a more unselected group of AF patients. Interestingly, a longitudinal study investigated a subset of patients with lone AF over 30  years, and it also found a subgroup of patients with a decrease in AF burden [20].

To date, risk factors for an increase or decrease in AF burden are largely unknown. It was hypothesized that AF either represents a primary electric disorder [26, 27] or reflects the consequence of other comorbidities including hypertension, obesity, and systemic, subclinical inflammation [28–30]. These different forms of AF might have a crucial influence on subsequent AF burden. We found that an increase in AF burden was independently associated with markers of left atrial disease, systolic and diastolic dysfunction, and a history of prior stroke. Our results, therefore, suggest an important role both of negative structural remodelling and cardiovascular risk factors in the change of AF burden over time. These findings add to the existing literature on the relation of structural left atrial disease with incident AF and with AF burden quantified by the clinical classification of AF [13, 18, 31]. Experimental animal studies underscore this concept, showing an increased susceptibility for AF in structurally abnormal left atria [9, 10]. Forming a vicious cycle, higher AF burden reciprocally seems to perpetuate this negative remodelling [32]. For maintaining this cycle, however, an underlying structural disease seems necessary [20, 32, 33]. In contrast, patients without overt structural heart disease, in which AF could be a primary electric disorder, might be more likely to develop a decrease in AF burden. These patients may still have a less diseased substrate that could be below the limit of detection of current diagnostic modalities. However, further studies are needed to provide more information on these relationships.

Some potential limitations have to be taken into account in the interpretation of our study. First, the observational nature of our study does not allow to draw causal inferences. Second, we may have missed episodes of AF due to the noncontinuous AF monitoring. However, the clinical significance of short AF episodes detected using more intense monitoring is still not known [21]. Third, we cannot exclude some regression to the mean in recorded AF burden over time. Fourth, patient drop out might not have been random and may have influenced our results. Fifth, changes in medication during follow-up might have influenced our results. Finally, the small number of participants limited our ability for multivariable adjustment and drawing of definite conclusions.

## 5. Conclusions

This prospective cohort study found that most patients with documented AF had no AF episodes recorded in yearly 7-day Holter-ECG recordings. A relevant portion of participants showed a decrease in AF burden over time. Overall, AF burden was independently associated with a history of prior stroke, BNP levels, left atrial size, and diastolic dysfunction suggesting an important role of structural heart disease. More studies are needed to better characterize the incidence of AF progression and predisposing risk factors for changes in AF burden over time.

## Figures and Tables

**Table 1:** Baseline characteristics stratified by the presence or absence of atrial fibrillation in Holter-ECG monitoring at the baseline study visit.

Characteristic	No AF burden (*n* = 29)	AF burden (*n* = 25)	*p*	Overall (*n* = 54)
Sex (male)	20 (69.0%)	19 (76.0%)	0.6	39 (72.2%)
Age, years	66.3 (9.2)	66.9 (7.0)	0.8	66.5 (8.2)
BMI, kg/m2	28.0 (4.8)	27.5 (5.2)	0.7	27.8 (4.9)
Time in AF per day, h	0 (0)	4.9 (7.1)	0.0006	2.3 (5.5)
Paroxysmal AF, n	29 (100%)	21 (84.0%)	0.04	50 (92.6%)
Medication				
Antiarrhythmic agent, n	9 (31.0%)	6 (24.0%)	0.8	15 (27.8%)
Beta-blocker, n	23 (79.3%)	13 (52.0%)	0.045	36 (66.7%)
ACE inhibitor or ARB, n	17 (58.6%)	10 (40.0%)	0.3	27 (50.0%)
Calcium-channel blocker, n	3 (10.3%)	4 (16.0%)	0.7	7 (13%)
Diuretic, n	6 (20.7%)	4 (16.0%)	0.7	10 (18.5%)
History of stroke, n	0 (0%)	7 (28.0%)	0.003	7 (13.0%)
History of CHF, n	3 (10.3%)	3 (12.0%)	0.99	6 (11.1%)
History of hypertension, n	20 (69.0%)	11 (44.0%)	0.1	31 (57.4%)
24h systolic BP, mmHg	121.1 (13)	117.8 (8.4)	0.3	119.5 (11.0)
CRP, mg/l	1.6 (0.8; 3.0)	1.9 (1.0; 3.6)	0.6	1.7 (0.9; 3.2)
BNP, pg/ml	54.7 (24.6; 111.7)	81.6 (42.6; 92.4)	0.4	60.2 (27.7; 94.4)
Left atrial diameter, mm	36.1 (7.0)	37.3 (7.3)	0.6	36.6 (7.1)
End-diastolic left atrial volume, ml	42.6 (13.2)	43.4 (16.9)	0.9	43.0 (14.8)
End-systolic left atrial volume, ml	69.4 (19.1)	66.9 (18.1)	0.7	68.3 (18.5)
Left atrial ejection fraction, %	38.8 (8.4)	36 (11.8)	0.4	37.6 (10.0)
Left atrial stroke volume, ml	26.8 (9.4)	23.6 (9.8)	0.3	25.3 (9.6)
Deceleration time, ms	228.4 (65.2)	221 (73.6)	0.7	225 (68.5)

Data are median (interquartile range), mean (standard deviation), or counts (percentage) as appropriate. *p* values are based on Fisher`s exact test, two independent samples *t*-test, or Wilcoxon as appropriate. AF = atrial fibrillation, ACE = angiotensin converting enzyme, ARB = angiotensin receptor blocker, BMI = body mass index, BNP = brain natriuretic peptide, BP = blood pressure, CHF = congestive heart failure, and CRP = C-reactive protein.

**Table 2:** Separate, mixed effect models for the relationships of atrial fibrillation burden and clinical, laboratory, and left atrial echocardiographic parameters.

	*β* (95% CI)	*p* value
BMI		
Crude	−0.33 (−1.67; 1.0)	0.6
Sex/Age-adjusted	−0.33 (−1.7; 1.04)	0.6

History of stroke		
Crude	42.59 (23.40; 61.77)	<0.0001
Sex/Age-adjusted	43.09 (23.22; 62.95)	<0.0001

History of CHF		
Crude	6.26 (−17.49; 30.02)	0.6
Sex/Age-adjusted	8.54 (−16.4; 33.48)	0.5

History of hypertension		
Crude	6.03 (−8.54; 20.59)	0.4
Sex/Age-adjusted	6.01 (−8.84; 20.86)	0.4

24 hour systolic BP		
Crude	−0.09 (−0.5; 0.32)	0.7
Sex/Age-adjusted	−0.09 (−0.5; 0.32)	0.7

CRP		
Crude	−0.84 (−2.23; 0.54)	0.2
Sex/Age-adjusted	−0.73 (−2.12; 0.66)	0.3

BNP		
Crude	0.05 (0.02; 0.09)	0.005
Sex/Age-adjusted	0.05 (0.02; 0.09)	0.005

Anterior-posterior diameter		
Crude	0.6 (−0.09; 1.29)	0.09
Sex/Age-adjusted	0.57 (−0.14; 1.28)	0.1

End-diastolic volume^*∗*^		
Crude	0.49 (0.23; 0.74)	0.0003
Sex/Age-adjusted	0.49 (0.23; 0.74)	0.0003

End-systolic volume^*∗*^		
Crude	0.25 (0.05; 0.46)	0.02
Sex/Age-adjusted	0.25 (0.05; 0.46)	0.02

Stroke volume^*∗*^		
Crude	−0.13 (−0.48; 0.22)	0.5
Sex/Age-adjusted	−0.13 (−0.49; 0.23)	0.5

Ejection fraction^*∗*^		
Crude	−0.43 (−0.76; −0.10)	0.01
Sex/Age-adjusted	−0.42 (−0.76; −0.09)	0.01

E-wave		
Crude	36.67 (12.96; 60.38)	0.003
Sex/Age-adjusted	37.93 (13.98; 61.89)	0.002

Deceleration time		
Crude	−0.1 (−0.16; −0.05)	0.0002
Sex/Age-adjusted	−0.1 (−0.16; −0.05)	0.0002

Data are *β* coefficients (95% confidence interval). Atrial fibrillation burden was used as the outcome variable. Participant identity numbers were computed as the random effect. Crude models included follow-up years as covariates and were further adjusted for sex and age. ^*∗*^3D echocardiographic parameter, BMI = body mass index, BNP = brain natriuretic peptide, BP = blood pressure, CHF = congestive heart failure, and CRP = C-reactive protein.

**Table 3:** Combined, mixed effect model for the relationships of atrial fibrillation burden and clinical, laboratory, and left atrial echocardiographic parameters.

	*β* (95% CI)	*p* value
Age	−0.31 (−1.1; 0.48)	0.4
Sex	−4.57 (−18.56; 9.41)	0.5
History of stroke	29.87 (2.61; 57.13)	0.03
BNP	0.05 (0.01; 0.08)	0.007
Ejection fraction^*∗*^	−0.2 (−0.65; 0.25)	0.4
Deceleration time	−0.06 (−0.13; 0.02)	0.1
E-wave	−1.45 (−35.15; 32.25)	0.9

Data are *β* coefficients (95% confidence interval). Atrial fibrillation burden was used as the outcome variable. Participant identity numbers were computed as the random effect. ^*∗*^3D echocardiographic parameter. BNP = brain natriuretic peptide.

## Data Availability

The data used to support the findings of this study are available from the corresponding author upon request.
